# Biodegradable nanoparticles are excellent vehicle for site directed *in-vivo *delivery of drugs and vaccines

**DOI:** 10.1186/1477-3155-9-55

**Published:** 2011-11-28

**Authors:** Anil Mahapatro, Dinesh K Singh

**Affiliations:** 1 Bioengineering Program & Department of Industrial and Manufacturing Engineering, Wichita State University, 1845 Fairmount Street, Wichita, KS 67260, USA; 2 Department of Life Sciences, Winston- Salem State University, 601 S MLK Jr. Drive Winston Salem, NC 27110, USA

**Keywords:** Biodegradable, nanoparticles, polyesters, vaccine delivery, drug delivery, gene delivery

## Abstract

Biodegradable nanoparticles (NPs) are gaining increased attention for their ability to serve as a viable carrier for site specific delivery of vaccines, genes, drugs and other biomolecules in the body. They offer enhanced biocompatibility, superior drug/vaccine encapsulation, and convenient release profiles for a number of drugs, vaccines and biomolecules to be used in a variety of applications in the field of medicine. In this manuscript, the methods of preparation of biodegradable NPs, different factors affecting optimal drug encapsulation, factors affecting drug release rates, various surface modifications of nanoparticles to enhance *in-vivo *circulation, distribution and multimodal functionalities along with the specific applications such as tumor targeting, oral delivery, and delivery of these particles to the central nervous system have been reviewed.

## Review

Nanotechnology, although not a new concept, has gained significant momentum in recent years. Due to the recent advances in material science and nano-engineering in the last decade, the nanoparticles have become very attractive for their applications in the fields of biology and medicine. Nanostructured materials are materials with sizes in the 1-100 nm range, which demonstrate unique properties and functions due to their "size effect"[1]. Since most biologically active macromolecules and agents such as viruses, membranes and protein complexes are natural nanostructures [2], it is assumed that nano-sized structures will be capable of enhanced interaction with cell membrane and proteins. The size and structure of nanoparticles also makes it easier for these materials to be integrated in to a number of biomedical devices. Within past few years, rapid developments have been made to use nanomaterials in a wide variety of applications in various fields of medicine such as cardiovascular and orthopedic. In medicine, nanomaterials have been used in specific applications such as tissue engineered scaffolds and devices, site specific drug delivery systems, cancer therapy and clinical bioanalytical diagnostics and therapeutics [3-5]. In recent years significant efforts have been made to use nanotechnology for the purpose of drug and vaccine delivery. The nanoparticles offer a suitable means to deliver small molecular weight drugs as well as macromolecules such as proteins, peptides or genes in the body using various routes of administration. The nano-sized materials provide a mechanism for local or site specific targeted delivery of macromolecules to the tissue/organ of interest, *in-vivo*. The newer developments in material science and nanoengineering are currently being leveraged to formulate therapeutic agents in biocompatible nanocomposites such as nanoparticles, nanocapsules, micellar systems and conjugates. In this manuscript, we have reviewed preparation of polymer based biodegradable nanoparticles and their applications in the field of medicine.

**Figure 1:**
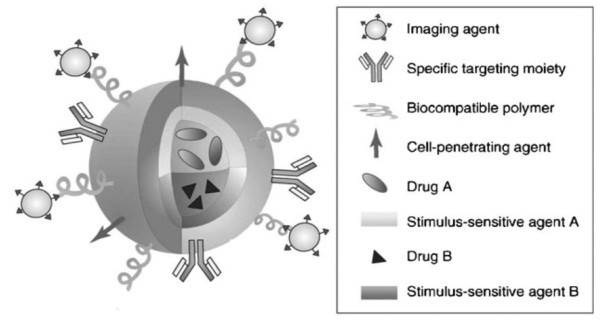
**Multifunctional nanoparticles**. Multifunctional nanoparticles can combine a specific targeting agent (usually with an antibody or peptide) with nanoparticles for imaging (such as quantum dots or magnetic nanoparticles), a cell-penetrating agent (e.g., the polyArg peptide TAT), a stimulus-selective element for drug release, a stabilizing polymer to ensure biocompatibility polyethylene glycol most frequently), and the therapeutic compound. Development of novel strategies for controlled released of drugs will provide nanoparticles with the capability to deliver two or more therapeutic agents. Adapted from ref [9] Copyright 2009 Wiley interscience.

Polymer-based nanoparticles are submicron-sized polymeric colloidal particles in which a therapeutic agent of interest can be embedded or encapsulated within their polymeric matrix or adsorbed or conjugated onto the surface [6]. These nanoparticles serve as an excellent vehicle for delivery of a number of biomolecules, drugs, genes and vaccines to the site of interest *in-vivo*. During the 1980's and 1990's several drug delivery systems were developed to improve the efficiency of drugs and minimize toxic side effects [7]. The early nanoparticles (NPs) and microparticles were mainly formulated from poly-alkyl-cyanoacrylate. The initial enthusiasm for the use of microparticles in medicine was later on dampened due to the size of the microparticles. There is a size limit for the particles to be able to cross the intestinal mucosal barrier of the gastrointestinal (GI) tract after the drug has been delivered orally. Most often, macroparticles could not cross mucosal barrier due to their bigger sizes resulting in failed delivery of drugs. Nanoparticles on the other hand have an advantage over microparticles due their nano-sizes. They are also better suited for intravenous (i.v.) delivery [8] compared to microparticles. Nanoparticles, however, had a different set of problems of their own. They had a very short circulating life span within the body after intravenous administration. The nanoparticles administered intravenously were rapidly cleared from the body by phagocytic cells. The therapeutic effect of drugs delivered via nanoparticles was thus minimized and could not be sustained. In recent years the problem of phagocytic removal of nanoparticles has been solved by surface modification of nanoparticles [7]. The surface modification protected nanoparticles from being phagocytosed and removed from the blood vascular system after intravenous injections. Now, a wide variety of biomolecules, vaccines and drugs can be delivered into the body using nanoparticulate carriers and a number of routes of delivery. NPs can be used to safely and reliably deliver hydrophilic drugs, hydrophobic drugs, proteins, vaccines, and other biological macromolecules in the body. They can be specifically designed for targeted drug delivery to the brain, arterial walls, lungs, tumor cells, liver, and spleen. They can also be designed for long-term systemic circulation within the body. In addition, nanoparticles tagged with imaging agents offer additional opportunities to exploit optical imaging or MRI in cancer diagnosis and guided hyperthermia therapy [9]. Figure 1 illustrates the possibility of using a multimodal approach and integrated systems that combine differing properties such as tumor targeting, cancer therapy and imaging in an-all-in one system [9]. Numerous techniques now exist for synthesizing different set of nanoparticles based on the type of drugs used, and the targeted organ and delivery mechanism selected. Depending upon the protocol of choice, the parameters can be tailored to create the best possible characteristics for the nanoparticles. In this manuscript we have reviewed a number of biodegradable nanoparticles currently in use, and the techniques of their preparation. We will also discuss advances in surface modifications, drug encapsulation and specific end applications of various types of NPs.

## Preparation of Nanoparticles

Biodegradable nanoparticles can be prepared from a variety of materials such as proteins, polysaccharides and synthetic biodegradable polymers. The selection of the base polymer is based on various designs and end application criteria. It depends on many factors such as 1) size of the desired nanoparticles, 2) properties of the drug (aqueous solubility, stability, etc.) to be encapsulated in the polymer, 3) surface characteristics and functionality, 4) degree of biodegradability and biocompatibility, and 5) drug release profile of the final product. Depending upon selection of desired criteria for the preparation of the nanoparticles, the methods can be classified as following 1) dispersion of preformed polymers, 2) polymerization of monomers and 3) ionic gelation method for hydrophilic polymers. The general advantages and disadvantages of individual methods are summarized in Table 1[10].

**Table 1:** Polymeric nanoparticles: general advantages and drawbacks of the various preparation methods (reproduced from ref [10] with permission from Elsevier).

Method	Simplicity of Procedure	Need for Purification	Facility Scaling-up	EE (%)	Safety of Compounds
**Polymerization of monomers**					

**Emulsion polymerization**					

Organic	Low	High	NR	Low	Low

Aqueous	High	High	High	High	Medium

Interfacial polymerization	Low	High	Medium	High	Low

**Preformed polymers**					

**Synthetic**					

Emulsification/solvent evaporation	High	Low	Low	Medium	Medium

Solvent displacement and interfacial deposition	High	NR	NR	High	Medium

Salting out	High	High	High	High	Low

Emulsion/solvent diffusion	Medium	Medium	High	High	Medium

**Natural**					

Albumin	NR	High	NR	Medium	Low

Gelatin	NR	High	NR	Medium	Low

**Polysaccharides**					

Alginate	High	Medium	High	High	High

Chitosan	High	Medium	High	High	High

Agarose	Medium	High	NR	NR	High

Desolvation	NR	High	NR	Low	Low

EE, Encapsulation Efficiency; NR, no reference available

### Dispersion of preformed polymers

This is the most commonly used technique to prepare biodegradable nanoparticles from poly-lactic acid (PLA); poly -D- L-glycolide (PLG); poly-D- L-lactide-co-glycolide (PLGA) and poly-cyanoacrylate (PCA). This technique can be used in several ways as described below.

#### (a) Solvent evaporation method

**Figure 2:**
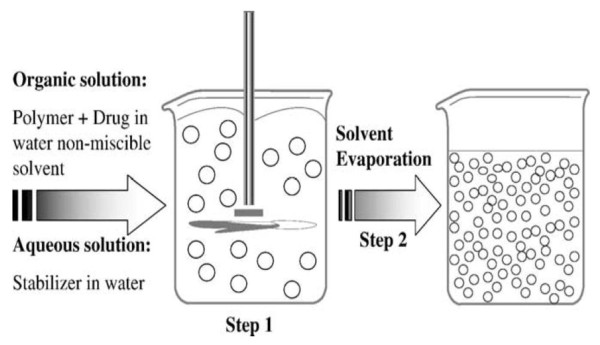
**Schematic representation of the emulsification-evaporation technique**. Adapted from ref [10] Copyright 2006 Elsevier.

In this technique the polymer is dissolved in an organic solvent such as dichloromethane, chloroform or ethyl acetate. The drug is dissolved or dispersed in the preformed polymer solution followed by emulsification of the mixture to form an oil/water (o/w) emulsion using an appropriate surfactant/emulsifying agents. Most commonly used surfactant/emulsifying agents for this purpose are gelatin and polyvinyl alcohol. After formation of a stable emulsion the organic solvent is evaporated by increasing the temperature or pressure along with continuous stirring of the solution. Figure 2 shows a schematic representation of this method [10]. Process parameters such as stabilizer and polymer concentration and stirring speed have a great influence on the particle size of the NPs formed [8,11].

#### (b) Spontaneous emulsification/solvent diffusion method

This is a modified solvent diffusion method where a water-miscible solvent such as acetone or methanol along with a water-insoluble organic solvent such as dichloromethane or chloroform are used as an oil phase [12]. Due to the spontaneous diffusion of solvents, an interfacial turbulence is created between the two phases leading to the formation of smaller particles. As the concentration of water- soluble solvent increases, smaller particle sizes of NPs can be achieved [10,12].

#### (c) Nanoprecipitation method

Typically, this method is used for hydrophobic drug entrapment, but it has been adapted for hydrophilic drugs as well. Polymers and drugs are dissolved in a polar, water-miscible solvent such as acetone, acetonitrile, ethanol, or methanol. The solution is then poured in a controlled manner (i.e. drop-by-drop addition) into an aqueous solution with surfactant. Nanoparticles are formed instantaneously by rapid solvent diffusion. Finally, the solvent is removed under reduced pressure [13].

#### (d) Salting out method

In this method, the polymer is dissolved in the organic phase, which should be water-miscible, like acetone or tetrahydrofuran (THF). The organic phase is emulsified in an aqueous phase, under strong mechanical shear stress. The aqueous phase contains the emulsifier and a high concentration of salts which are not soluble in the organic phase. Typically, the salts used are 60% w/w of magnesium chloride hexahydrate [14] or magnesium acetate tetrahydrate in 1:3 polymer to salt ratio [15]. Contrary to the emulsion diffusion method, there is no diffusion of the solvent due to the presence of salts. The fast addition of pure water to the o/w emulsion under mild stirring reduces the ionic strength and leads to the migration of the water-soluble organic solvent to the aqueous phase inducing nanosphere formation. The final step is purification of nanoparticles by cross flow filtration or centrifugation to remove the salting out agent [14,15].

### Polymerization Methods

NPs are prepared from monomers that are polymerized to form NPs in an aqueous solution. Vaccines or drugs/therapeutic agents are incorporated in the NPs either by dissolving the drug in the polymerization medium or by adsorption/attachment of the drug onto the polymerized and fully formed NPs. The NP suspension is then purified by removing stabilizers. The surfactants may be recycled for subsequent polymerization. This technique of NPs preparation has been reported for making polybutylcyanoacrylate or poly-alkyl-cyanoacrylate NPs [16,17]. The concentration of surfactant and the stabilizer determines the final size of the NPs formed [18].

### Ionic gelation method for hydrophilic polymers

Some of the natural macromolecules have been used to prepare NPs. These polymers include gelatin, alginate, chitosan and agarose. They are hydrophilic natural polymers and have been used to synthesize biodegradable NPs by the ionic gelation method. This involves the transition of materials from liquid to gel due to ionic interaction at room temperature. An example of preparation of gelatin NPs includes hardening of the droplets of emulsified gelatin solution into gelatin NPs. The gelatin emulsion droplets are cooled below the gelation point in an ice bath leading to gelation of the droplets [19] into gelatin NPs. Alginate NPs are reported to be produced by drop-by-drop extrusion of the sodium alginate solution into the calcium chloride solution [20]. Sodium alginate is a water-soluble polymer that gels in the presence of multivalent cations such as calcium [21]. Chitosan NPs are prepared by spontaneous formation of complexes between chitosan and polyanions or by the gelation of a chitosan solution dispersed in an oil emulsion [22].

## Biodegradable Nanoparticles

Biodegradable nanoparticles have been used for site-specific delivery of drugs, vaccines and various other biomolecules. A few of the most extensively used biodegradable polymer matrices for preparation of nanoparticles are:

### Poly-D-L- lactide-co-glycolide (PLGA)

**Figure 3:**
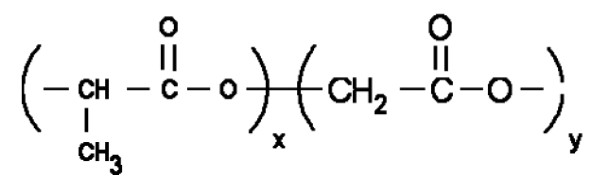
**Structure of PLGA**. The suffixes x and y represent the number of lactic and glycolic acid respectively.

Poly-D-L- lactide-co-glycolide (PLGA) is one of the most successfully used biodegradable polymers. It undergoes hydrolysis in the body to produce biodegradable metabolite monomers such as lactic acid and glycolic acid. Figure 3 depicts the schematic representation of the chemical structure of PLGA. Since lactic acid and glycolic acids are normally found in the body and participate in a number of physiological and biochemical pathways, there is very minimal systemic toxicity associated with the use of PLGA for the drug delivery or biomaterial applications. PLGA NPs have been mostly prepared by the emulsification-diffusion, the solvent evaporation and the nanoprecipitation methods [23]. PLGA nanoparticles have been used to develop protein and peptide based nanomedicines, nano-vaccines, and genes containing nanoparticles for *in-vivo *delivery systems [23,24].

### Polylactic acid (PLA)

**Figure 4:**
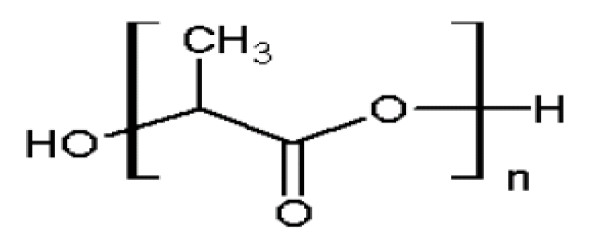
**Chemical structure of poly lactic acid (PLA)**.

PLA (Figure 4) is a biocompatible and biodegradable polymer which is broken down to monomeric units of lactic acid in the body. Lactic acid is a natural intermediate/by product of anaerobic respiration, which is converted into glucose by the liver during the Cori cycle. Glucose then is used as an energy source in the body. The use of PLA nanoparticles is therefore safe and devoid of any major toxicity. PLA nanoparticles have been mostly prepared by the solvent evaporation, solvent displacement, salting out and solvent diffusion methods [10,25]. The salting out procedure is based on the separation of a water- miscible solvent from aqueous solution by adding a salting out agent like magnesium chloride or calcium chloride. The main advantage of the salting out procedure is that it minimizes stress to protein encapsulants [23].

### Poly-ε-caprolactone (PCL)

**Figure 5:**
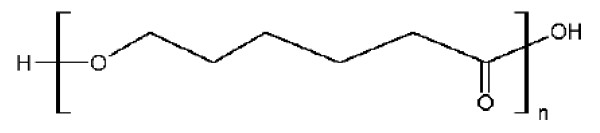
**Chemical structure of Poly-ε-caprolactone (PCL)**.

poly-ε-caprolactone (Figure 5) is degraded by hydrolysis of its ester linkages under the normal physiological conditions in the human body and has minimal or no toxicity. Therefore, PCL has grabbed the attention of researchers as a candidate of choice for use in drug delivery and long-term implantable devices. PCL's slower rate of degradation compared to polylactides has made it better candidate for making long-term implantable devices. PCL nanoparticles have been prepared mostly by nanoprecipitation, solvent displacement and solvent evaporation [23,26,27].

### Chitosan

**Figure 6:**
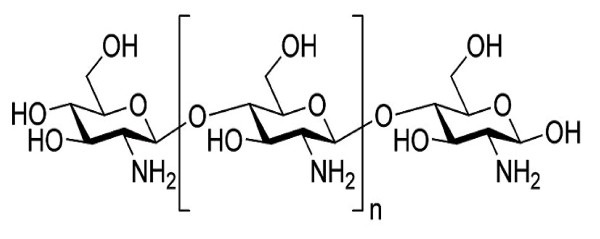
**Chemical structure of chitosan**.

Chitosan (Figure 6) is a modified natural carbohydrate polymer prepared by the partial N-deacetylation of the crustacean-derived natural biopolymer chitin. There are at least four methods reported for the preparation of chitosan nanoparticles. The four methods are ionotropic gelation, microemulsion, emulsification solvent diffusion and polyelectrolyte complex formation [23,28,29].

### Gelatin

**Figure 7:**
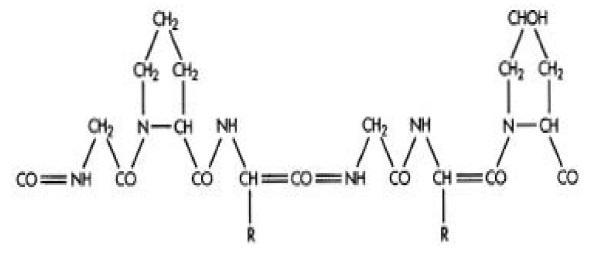
**Chemical structure of Gelatin**.

Gelatin (Figure 7) is extensively used in food and medical products and is a nontoxic alternative. Gelatin NPs are very efficient in delivery and controlled release of the drugs. They are nontoxic, biodegradable, bioactive and inexpensive. Gelatin is a poly-ampholyte consisting of both cationic and anionic groups along with a hydrophilic group. It is known that the mechanical properties such as swelling behavior and thermal properties of gelatin NPs depend significantly on the degree of cross-linking between cationic and anionic groups. These properties of gelatin can be manipulated to prepare desired type of NPs from gelatin. Gelatin nanoparticles can be prepared by the desolvation/coacervation or emulsion methods [23,30,31].

#### Poly-alkyl-cyano-acrylates (PAC)

**Figure 8:**
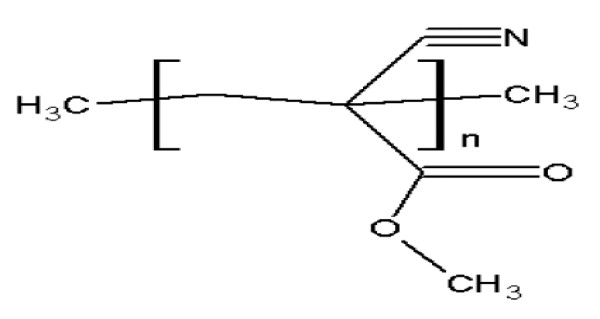
**Chemical structure of *Poly-alkyl-cyano-acrylates***.

The biodegradable as well as biocompatible poly-alkylcyanoacrylates (Figure 8) are degraded by enzyme esterases found in the body. On degradation they produce some toxic products that may stimulate or damage the central nervous system. Thus this polymer is not authorized for application in humans. PAC nanoparticles are prepared mostly by emulsion polymerization, interfacial polymerization and nanoprecipitation [10,23].

## Surface Modification

One of the problems faced in the use of nanoparticles via the intravenous route was their speedy removal by the phagocytic cells (macrophages) in the body. Macrophages are powerful phagocytic cells and are the important constituent of mononuclear phagocytic system (MPS). The mononuclear phagocytic system (MPS) is one of the body's innate defenses. MPS filters and eliminates any injected particulate matter including NPs from the blood stream if they are recogniozed as foreign body. Unless the injected nanoparticles are modified in a way to escape recognition as foreign particles, they will be phagocytosed and removed from the circulation. This necessitated modification of the surface of nanoparticles in order for them to escape MPS recognition and subsequent clearance. Surface modification of the NPs therefore plays a critical role in their successful applications *in-vivo *[32]. Once NPs are surface modified with biomolecules found normally in the body, they will be able to circulate within the blood vascular system for longer period of time. This increases the probability of nanoparticles reaching their target rapidally and safely when compared to non- modified NPs. Smaller particles (< 100 nm) circulating in blood vascular system with a hydrophilic surface have the greatest ability to evade the MPS [33,34]. Several methods have been developed for surface modification of the NPs. The most preferred method of surface modification is the adsorption or grafting of poly-ethylene glycol (PEG) to the surface of nanoparticles. Addition of PEG and PEG-containing copolymers to the surface of nanoparticles results in an increase in the blood circulation half-life of the particles. The exact mechanisms by which PEG prolonged circulation time of the surface modified NPs are still not well understood. It is generally thought that the increased residency of the nanoparticles in blood is mainly due to prevention of opsonization of nanoparticles by a certain serum or plasma proteins (opsonins). It is believed that PEG causes steric repulsion by creating hydrated barriers on nanoparticle surfaces that prevents coating of PEG modified NPs by serum opsonins.

Studies have shown that the degree to which proteins (opsonins) adsorb onto particle surface can be minimized by increasing the PEG density on the particle surface. Increasing the molecular weight of the PEG chains [33] has also been shown to minimize opsonization of nanoparticles and improve retention in the circulation. For example, Leroux et al. [35] showed that an increase in PEG molecular weight was associated with less interaction with the MPS, and longer systemic circulation of PLGA nanoparticles. PEG has been shown to impart stability on PLA particles submerged in simulated gastric fluid (SGF). Tobio et al. [36] showed that after 4 hours in SGF, 9% of PLA nanoparticles converted to lactic acid versus 3% conversion for PEG-PLA particles [36]. PEG is also believed to facilitate mucoadhesion and consequent transport through the Peyer's patches of the GALT (gut associated lymphoid tissue) [37]. In addition, PEG may benefit nanoparticle's interaction with blood constituents. Thus, the presence of PEG on the nanoparticles imparts additional functionality during the use of polymeric NPs.

Apart from PEG, there are other hydrophilic polymers such as poloxamers, polysorbate 80, TPGS, polysorbate 20, polysaccharides like dextran and different type of copolymers that can be used to efficiently coat conventional nanoparticles to add number of variations in the surface properties of NPs [38,39]. These coatings provide a dynamic cloud of hydrophilic and neutral chains at the particle surface, which repels plasma proteins. Surface modification by TPGS increases the adhesion of nanoparticles to tumor cell's surfaces. It also provides safer environments to the encapsulated proteins. IgG coating on the surface of nanoparticles increases the immunoresponse to the encapsulated proteins within the nanoparticles. Hydrophilic polymers can be applied at the surface of NPs by adsorption of surfactants or by use of block copolymers or branched copolymers [38-40].

## Drug Loading and Encapsulation

One of the most desired qualities of a successful nanoparticle is its high loading capacity for the drugs. The high loading ability of NPs reduces the amount of the polymer carrier required for vaccine/drug delivery in the body. The loading of drugs/vaccine into/onto nanoparticles is achieved by two methods: 1) by incorporating the drug at the time of nanoparticle production or 2) by adsorbing the drug after the formation of nanoparticles. Adsorption of drugs is achieved by incubating the NPs in a concentrated drug solution [8]. These two methods provide number of ways by which the drug is adsorbed/attached to the NPs. The encapsulation of the drug in the polymer, dispersion of the drug in the polymer, adsorption of the drug onto the surface of the nanoparticles and chemical binding of the drug to the polymer can be accomplished using incorporation/adsorption techniques. The amount of drugs bound to NPs and the type of interaction -between drugs and nanoparticles depend on the chemical structure of the drug, chemical structure of the polymer and the conditions of drug loading [41]. The amount of bound drug can be determined by subtracting the drug content in the supernatant from the primary amount of drug present in the suspension.

The drug release mechanisms are an equally important consideration during drug polymer formulation. It will influence the effectiveness of the proposed application and successful sustained drug delivery. In general, the drug release rate depends on solubility of the drug, desorption of the surface bound/adsorbed drug, drug diffusion through the polymer matrix, NP matrix erosion/degradation and combination of the erosion diffusion process [23]. For manipulation of the drug release, a good understanding of the mechanisms of drug release is needed which would involve knowledge of the solubility, diffusion and biodegradation of the matrix. One way to modify the drug release profile is by adopting appropriate polymer matrices. Drug release kinetics also depend upon size of the NPs and the loading efficiency of the vaccine or drug. The vaccine or drug loading efficiency will determine the initial burst and the sustained release rate of nanoencapsulated drug molecule. Larger particles have a smaller initial burst release than smaller particles. In the case of nanospheres, where the vaccine/drug is uniformly distributed, the release occurs by diffusion or erosion of the matrix under sink conditions. If the diffusion of a vaccine/drug is faster than the matrix erosion, the release mechanism is predominately through a diffusion process. The rapid initial release or burst of vaccine/drug seen in release profiles is mainly attributed to weakly bound or adsorbed vaccine/drug on to the surface [7,42].

## Specific Applications of Biodegradable NPs

### Tumor Targeting

The rationale of using nanoparticles for tumor targeting is based on 1) NP's ability to deliver the requisite dose load of drug in the vicinity of the tumor due to the enhanced permeability and retention effect or active targeting by ligands on the surface of NPs and 2) NP's ability to reduce the drug exposure to healthy tissues by limiting drug distribution to the target organ. Active tumor targeting of NPs may be achieved with either direct targeting or the pretargeting method. In direct targeting method NPs are covalently coupled with the ligands. The ligand coupled NPs are received by the tumor cells expressing a homologous receptor on their surfaces. The specific ligand-receptor binding ensures that the NPs carrying drugs will get attached specifically to the tumor cells. This will facilitate delivery of drugs only to the cells (tumor cells) expressing receptor and not the normal healthy cells. In the pretargeting approach, the therapeutic molecule is not coupled with the ligand and is administered after an appropriate delay time following the administration of the targeting ligand. Nobs et al. [43] explored both-approaches to target PLA NPs to tumor cells. In the direct approach, NPs with mAbs exposed on their surface were incubated with the two tumor cells, while in the pretargeting protocol, tumor cells were pretargeted with biotinylated MABs prior to the administration of avidin-labelled NPs [43].

Verdun et al. [44] in an elegant experiment demonstrated positive effects of using poly- isohexylcyanoacrylate-nanospheres in the delivery of doxorubicin in mice. The doxorubicin incorporated into poly (isohexylcyanoacrylate) nanopsheres and delivered in mice showed higher concentrations of doxorubicin in the liver, spleen and the lungs than in mice treated with only free doxorubicin [44]. Studies show that the drug distribution pattern in the body is greatly influenced by selected drug's molecular weight, polymeric composition (type, hydrophobicity and biodegradation profile) of nanoparticles, localization of drug in the nanospheres, and drug incorporation techniques such as adsorption or incorporation [45].

Extensive efforts have been devoted to achieving "active targeting" of nanoparticles in order to deliver drugs to the right targets. The molecular recognition processes such as ligand-receptor specificity or antigen-antibody interaction plays important role in such targeting. Considering that folate receptors are over expressed on the surface of some human malignant cells and that cell adhesion molecules such as selectins and integrins are involved in metastatic events, nanoparticles bearing specific ligands such as folate may be used to target ovarian carcinoma while specific peptides or carbohydrates may be used to target integrins and selectins [46]. Oyewumi et al. [47] demonstrated that the benefits of folate ligand coating were to facilitate internalization and retention of Gd-nanoparticles in the tumor cells/tissues [47]. Targeting with small ligands appears more likely to succeed since they are easier to handle and manufacture. Furthermore, it could be advantageous to use active targeting ligands in combination with the long-circulating nanoparticles to maximize the likelihood of active targeting of nanoparticles.

### Nanoparticles for Oral delivery

In recent years, significant research has been done using nanoparticles as oral drug delivery vehicles. Oral delivery of drugs using nanoparticles has been shown to be far superior to the delivery of free drugs in terms of bioavialability, residence time, and biodistribution [48]. Advances in biotechnology and biochemistry have led to the discovery of a large number of bioactive molecules and vaccines based on peptides and proteins. Development of suitable carriers remains a challenge due to the fact that bioavailability of these molecules is limited by the epithelial barriers of the gastrointestinal tract. The drugs may also be susceptible to gastrointestinal degradation by digestive enzymes. The advantage of using polymeric nanoparticles is to allow encapsulation of bioactive molecules and protect them against enzymatic and hydrolytic degradation. For instance, it has been found that insulin-loaded nanoparticles have preserved insulin activity and produced blood glucose reduction in diabetic rats for up to 14 days following the oral administration [49].

Another study showed that an antifungal drug encapsulated in particles of less than 300 nm in diameter was detected in the lungs, liver, and spleen of mice seven days post oral administration. The oral-free formulations on the other hand were cleared within 3 hours post administration [48]. For this application, the major interest lies in lymphatic uptake of the nanoparticles by the Peyer's patches in the GALT (gut associated lymphoid tissue). There have been many reports as to the optimum size for Peyer's patch uptake ranging from less than 1 μm to 5 μm [50,51]. However, it has also been shown that microparticles remain in the Peyer's Patches while nanoparticles are disseminated systemically [52,53]

Nanoparticles can be engineered not only for oral absorption, but can also be used to deliver a drug directly to the source for gastrointestinal uptake, thereby protecting the drug from low pH and enzymes in the stomach. The pH-sensitive nanoparticles made from a poly(methylacrylic acid and methacyrlate) copolymer can increase the oral bioavailability of drugs like cyclosporine-A by releasing their load at a specific pH within the gastrointestinal tract. The pH sensitivity allows this to happen as close as possible to the drug's absorption window through the Peyer's patches [54].

### Nanoparticles for vaccine/gene delivery

Polynucleotide vaccines/DNA vaccines/plasmid vaccines work by delivering genes encoding relevant antigens to host cells where they are expressed, producing the antigenic protein within the vicinity of professional antigen presenting cells to initiate immune response. Such vaccines produce both humoral and cell-mediated immunity because intracellular production of protein, as opposed to extracellular deposition, stimulates both arms of the immune system [55]. The key ingredient of polynucleotide vaccines, DNA, can be produced cheaply and has much better storage and handling properties than the ingredients of the majority of protein-based vaccines. Hence, polynucleotide vaccines/DNA vaccines are set to supersede many conventional vaccines particularly for immunotherapy. However, there are several issues related to the delivery of polynucleotides which limit their application. These issues include efficient delivery of the polynucleotide to the target cell population, its localization to the nucleus of these cells, and ensuring that the integrity of the polynucleotides is maintained during delivery to the target site [2]. Nanoparticles loaded with plasmid DNA could also serve as an efficient sustained release gene delivery system due to their rapid escape from the degradative endo-lysosomal compartment to the cytoplasmic compartment [56]. Hedley et al. [57] reported that following their intracellular uptake and endolysosomal escape, nanoparticles could release DNA at a sustained rate resulting in continuous gene expression. This gene delivery strategy could be applied to facilitate bone healing by using PLGA nanoparticles containing therapeutic genes such as bone morphogenic protein.

### Nanoparticles for drug delivery into the brain

The blood-brain barrier (BBB) is the most important factor limiting the development of new drugs for the central nervous system [58]. The BBB is characterized by relatively impermeable endothelial cells with tight junctions, enzymatic activity and active efflux transport systems. It effectively prevents the passage of water-soluble molecules from the blood circulation into the CNS, and consequently only permits selective transport of molecules that are essential for brain function [59]. Strategies for nanoparticle targeting to the brain rely on nanoparticle's interaction with the specific receptor-mediated transport systems in the BBB. For example, polysorbate 80/LDL, transferrin receptor binding antibody (such as OX26), lactoferrin, cell penetrating peptides and melanotransferrin have been shown to be capable of delivery of a self non transportable drug into the brain via the chimeric construct that can undergo receptor-mediated transcytosis [60-63]. It has been reported that poly(butylcyanoacrylate) nanoparticles were able to deliver hexapeptide dalargin, doxorubicin and other agents into the brain which is significant because of the great difficulty for drugs to cross the BBB [62]. Despite some reported success with polysorbate 80 coated NPs, this system does have many shortcomings including desorption of polysorbate coating, rapid NP degradation and toxicity caused by presence of high concentration of polysorbate 80 [64]. OX26 MAbs (anti-transferrin receptor MAbs), the most studied BBB targeting antibody, have been used to enhance the BBB penetration of lipsosomes [65].

Another study by Kreuter et al. [66] demonstrates the delivery of several drugs successfully through the blood brain barrier using polysorbate 80 coated PACA nanoparticles [66]. It is thought that after administration of the polysorbate 80-coated particles, apolipoprotein E (ApoE) adsorbs onto the surface. The ApoE protein mimics low density lipoprotein (LDL) causing the particles to be transported across the blood brain barrier via the LDL receptors. The effects of polysorbate-80 on transport through the blood brain barrier were confirmed by Sun et al. with PLA nanoparticles [67]. Nanoparticles were also functionalized with thiamine surface ligands. These particles, with an average diameter of 67 nm, were able to associate with the blood brain barrier thiamine transporters and thereby increase the unidirectional transfer coefficient for the particles into the brain [68].

## Conclusion

In summary, NPs are a potentially viable vaccine and drug delivery system capable of delivering a multitude of therapeutic agents and biomolecules at the targeted sites in the body. To optimize NPs as a delivery system, greater understanding of the different mechanisms of biological interactions and particle engineering is still required. However, biodegradable NPs appear to be a promising drug delivery carrier system because of their versatile formulation, sustained release properties, sub cellular size and biocompatibility with various cells and tissue in the body.

## List of Abbreviations Used

NPs: Nanoparticles; PLA: Poly-lactic acid; PLG: poly (D; L-glycolide); PLGA: Poly (D; L-lactide-co-glycolide; PCA: Poly-cyanoacrylate; THF: Tetrahydrofuran; PCL: Poly-ε-caprolactone; PAC: Poly-alkyl-cyano-acrylate; MPS: Mononuclear Phagocytic System; PEG: Poly-ethylene glycol; SGF: Simulated gastric fluid; GALT: Gut-associated lymphoid system; TPGS: Tocopheryl polyethylene glycol 1000 succinate; mABs: Monoclonal antibodies; BBB: Blood-brain barrier; ApoE: Apolipoprotein E; LDL: low density lipoprotein.

## Competing interests

The authors declare that they have no competing interests.

## Authors' contributions

Both authors have read and approved the final manuscript. AM participated in conceptualization and preparation of this manuscript. He contributed in preparation of nanoparticles, biodegradable nanoparticles and surface modification of nanoparticles sections of this manuscript. DKS participated in specific application of biodegradable NPs section. DKS also participated in the conceptualization of the manuscript, writing, editing and revision of this report. His lab provided materials and resources used in this study.

## Authors information

AM: is an assistant professor of Bioengineering in the Department of Industrial and Manufacturing Engineering at Wichita State University, Wichita, KS. AM's lab is working on development and application of biodegradable implants and drug delivery systems. AM's lab is developing biodegradable nanoparticles for gene and drug delivery and is also participating in a collaborative project with DKS' lab on the use of biodegradable nanoparticles in delivering a HIV- DNA vaccine within the cervical and vaginal mucosa.

DKS: is an associate professor of microbiology at the Winston Salem State University. DKS' lab is working on development of a DNA vaccine for HIV/AIDS. His other research interest involves prevention of HIV-1 transmission at the cervical/vaginal mucosal surfaces, use of nanoparticles in preventing transmission of HIV at the mucosal surfaces. DKS is also participating in a collaborative project with AMS' lab on the use of biodegradable nanoparticles in delivering a HIV- DNA vaccine within the cervical and vaginal mucosa. His current research is funded by two NIH grants.
